# The Assessment of Metacognition in Psychosis: Systematic Review and Future Lines of Research

**DOI:** 10.1002/cpp.70223

**Published:** 2026-02-08

**Authors:** Luciana Díaz‐Cutraro, Marina Verdaguer‐Rodriguez, Marta Ferrer‐Quintero, Roger Montserrat, Steffen Moritz, Paul Lysaker, Giancarlo Dimaggio, Carolina Palma‐Sevillano, María Lamarca, Victoria Espinosa, Rabea Fischer, Marina Peniza‐Soriano, Raquel López‐Carrilero, Helena García‐Mieres, Susana Ochoa

**Affiliations:** ^1^ Fundació de Recerca Sant Joan de Déu Barcelona Catalonia Spain; ^2^ Grup MERITT Institut de Recerca Sant Joan de Déu Barcelona Catalonia Spain; ^3^ Unitat de Recerca Parc Sanitari Sant Joan de Déu Barcelona Catalonia Spain; ^4^ Grup COMSAL Blanquerna FPCEE – Universitat Ramon Llull Barcelona Catalonia Spain; ^5^ Department of Clinical and Health Psychology, Faculty of Psychology Universitat Autònoma de Barcelona Barcelona Spain; ^6^ Department of Child and Adolescent Psychiatry, Institute of Psychiatry and Mental Health, Hospital General Universitario Gregorio Marañón, IiSGM, CIBERSAM, School of Medicine Universidad Complutense Madrid Spain; ^7^ Facultat de Psicologia Universitat de Barcelona Barcelona Spain; ^8^ Clinical Neuropsychology Working Group, Department of Psychiatry and Psychotherapy University Medical Center Hamburg‐Eppendorf Hamburg Germany; ^9^ Department of Psychiatry Indiana University School of Medicine Indianapolis Indiana USA; ^10^ Centro di Terapia Metacognitiva Interpersonale Rome Italy; ^11^ Hospital de Mataró Maresme Sanitary Consortium Barcelona Catalonia Spain; ^12^ Consorcio de Investigación Biomédica en Red de Salud Mental (CIBERSAM) Instituto de Salud Carlos III Madrid Spain; ^13^ Universitat Oberta de Catalunya (UOC) Barcelona Spain

**Keywords:** clinical assessment, clinician‐reported measures, metacognition–assessment–metacognitive model, patient‐reported measures, research assessment

## Abstract

Impaired metacognition, the capacity to understand one's own and others' mental states, has gained increasing attention in psychosis research. Different conceptualizations, psychological treatments and assessment methods have emerged; however, there is a lack of consensus regarding the appropriate tools for clinical and research use. This systematic review had two aims: (1) to compile and organize available assessment tools and (2) to propose an index of metacognitive domains and processes. Instruments were categorized according to authorship, year of use in psychosis, outcomes assessed, language/version, administration time and type of Clinical Outcome Assessment (ClinRO, PRO or PerfO). We identified 42 studies that used 31 instruments. The tools were classified into four domains: Metacognitive Awareness, Metacognitive Capacity, Neurometacognition and Social Metacognition. Our findings highlight the diversity of the available measures and propose a framework for aligning instruments with specific reflective processes. This work represents a practical and theoretical first step toward building consensus and facilitating both the use of available tools according to practical needs and the development of an agreed‐upon definition and components of metacognition.

## Summary

- Metacognition is the capacity to reflect on one's own and others' psychological processes, including thoughts, emotions, behaviours and relationships.
- Deficits in metacognition are robustly associated with poorer functional outcomes in psychosis, even when controlling for symptom severity and neurocognition.
- More than 30 instruments have been detected to assess metacognition in psychosis, but the lack of consensus makes their clinical use and research comparisons difficult.
- We propose a framework grouping these tools into four main areas—Metacognitive Awareness, Metacognitive Capacity, Neurometacognition and Social Metacognition—making it easier to select instruments depending on the type of reflective processes of interest.
- In practice, short self‐report scales can be useful in busy clinical settings, while interviews or performance‐based tasks are more suitable for research or specialized care.

## 1 Introduction

### 1.1 Definitions of Metacognition

Metacognition has been conceptualized in various ways (Flavell 1979; Frith 2017; P. H. Lysaker et al. 2018; Moritz et al. 2019; Moritz and Lysaker 2018b; Quiles et al. 2020; Reeder et al. 2010; Sellers, Wells, Parker, and Morrison 2018). Despite these different definitions, a common denominator is the understanding of metacognition at a supraordinate level encompassing distinct domains and functions related to thinking, emotions and behaviours.

In 1979, Flavell (1979) theorized that metacognition is linked to learning in children and suggested transferring this knowledge to the field of psychological therapy. He posited metacognition as a process that supervises or monitors cognitive content through four subprocesses: metacognitive knowledge, metacognitive experiences, goals or tasks and actions or strategies. The interaction between these four pillars allows us to move from merely modifying cognitive knowledge to becoming aware of how that knowledge has been formed, whether it is accurate, whether it is goal‐related and how to improve it through motivation, if necessary. For these reasons, Flavell defined metacognition as thinking or reflecting on one's thoughts.

In the context of schizophrenia, several recent studies have synthesized key findings and theoretical developments (Brüne 2014; Cella et al. 2015; Kircher et al. 2007; P. H. Lysaker et al. 2021; Moritz and Lysaker 2018b; Morrison et al. 2007; Morrison and Wells 2007; Philipp et al. 2020; A. Pinkham 2018; A. E. Pinkham 2019; Sellers, Wells, and Morrison 2018; Wright et al. 2021). At one end of this continuum lie discrete processes such as awareness of the accuracy of one's own judgements or reasoning. At the other end, there are more integrative and synthetic processes through which individuals organize information about themselves and others into coherent representations of identity, intentions and emotions (P. H. Lysaker et al. 2021; A. Pinkham 2018). This spectrum‐based perspective helps to explain the diversity of metacognitive constructs and measures found in the literature, from task‐specific assessments to broader conceptualizations involving the self.

Semerari et al. proposed an influential model that conceptualizes metacognition as a set of semi‐independent abilities. These include awareness of one's own mental states (e.g., thoughts and affects), recognition of their mutual influence on behaviour, understanding of others' mental states and the capacity to acknowledge the subjective nature of one's own ideas. In this framework, metacognition also includes mastery or the ability to use this knowledge to reduce distress and solve interpersonal or psychological problems (Carcione et al. 2011; Semerari et al. 2003).

A slightly different perspective concerns the interaction between metacognition and the cognitive processes. For example, awareness of one's performance in cognitive tasks involves regulatory processes that guide learning and information processing by modulating the allocation of cognitive resources. Individuals with schizophrenia often present a metacognitive mismatch, meaning that there is a discrepancy between subjective estimations and actual performance in cognitive tasks, manifesting as either an underestimation of difficulties or overconfidence in one's skills (Cella et al. 2015).

The importance of studying metacognition lies in its strong association with functional outcomes in psychosis even after accounting for symptom severity and cognitive deficits (Van Oosterhout et al. 2016). Consequently, several therapeutic approaches have been developed to address deficits or distortions in metacognitive functioning. Psychological interventions targeting metacognition have shown promising results in reducing symptoms and cognitive biases while improving the overall quality of life (Inchausti et al. 2017, 2018; Moritz et al., n.d.; Moritz et al. 2014; Moritz and Lysaker 2018a; Morrison et al. 2014; A. Pinkham 2018).

#### 1.1.1 Metacognition as a Meta‐Level

Given the conceptual diversity and lack of consensus in this field, it is useful to clarify the integrative definition of metacognition adopted in this study. We focused on studies that considered metacognition as a superordinate, higher order process—one that involves thinking about one's own or others' psychological functioning (Nelson 1990; Nelson et al. 1999).

The terminology used in the literature to refer to higher‐order processes includes *metacognition*, *cognitive insight* (Beck et al. 2004), *self‐regulatory executive function*, *thought suppression* (Nittel et al. 2018), meta‐thinking (Ackerman and Thompson 2017), reflective function (De Jong et al. 2019), *conscientiousness* and *self‐reflectiveness*. Although not exhaustive, this list illustrates the various ways in which metacognitive processes in psychosis have been described.

In recent years, the research and therapeutic interest in metacognition in psychosis has grown significantly. Different therapies incorporate metacognitive components, each within a distinct framework. Philipp et al. (2020) provided an overview of three main approaches targeting metacognition: Metacognitive Therapy (MCT), Metacognitive Training and Metacognitive Reflection and Insight Therapy (MERIT). Although Metacognitive Therapy and MERIT are formal therapeutic models, Metacognitive Training is a structured group‐based intervention designed to raise awareness of cognitive biases and enhance metacognitive skills in individuals with psychosis.

Metacognitive Therapy is a transdiagnostic approach that focuses on beliefs about cognition and strategies used to regulate thoughts and attention. It aims to reduce the cognitive attentional syndrome by promoting more adaptive metacognitive strategies (Hutton et al. 2014; Morrison et al. 2014). Metacognitive Training is a structured, group‐based intervention developed to increase awareness of common cognitive biases that contribute to the development and maintenance of symptoms across various mental disorders (Kühl et al. 2021; Moritz et al. 2011, 2013, 2014; Van Oosterhout et al. 2016). Offered in diverse formats (e.g., group, family, individual or crisis‐adapted), it encourages reflection and the generation of alternative thinking strategies, ultimately aiming to foster long‐term metacognitive awareness of cognitive distortions, particularly in psychosis (Moritz et al. 2014).

Metacognitive Reflection and Insight Therapy (MERIT) focuses on developing a more integrated understanding of one's own and others' mental states (De Jong et al. 2019). Drawing on the therapeutic relationship that promotes reflection, MERIT conceptualizes metacognition as a set of related but semi‐independent abilities. This includes awareness of cognitive and emotional states, understanding their influence on behaviour, recognizing the subjectivity of one's own views, and using this awareness to cope with challenges and enhance interpersonal functioning (Lysaker et al. 2016). In addition, MERIT explicitly promotes the capacity to reflect not only on the cognitive and emotional states of self and others but also on the broader social context, supporting a movement from centrality to decentration. Additional approaches have also integrated metacognitive components into their frameworks. These include Metacognition‐Oriented Social Skills Training (Ottavi et al. 2014), evolutionary systems therapy (Cheli et al. 2023) and recovery‐focused metacognitive interpersonal therapy (Inchausti et al. 2023). Despite their differences in theoretical background and method of delivery, all these approaches share a common goal: to increase awareness of psychological states and foster more adaptive self‐regulatory strategies. This, in turn, is expected to help individuals with psychosis better understand themselves and others, navigate social difficulties and pursue personal needs and aspirations (Dimaggio et al. 2015; Philipp et al. 2020).

### 1.2 Assessment of Metacognition in Psychosis: State of the Art and Present Study Proposal

Along with the variety of definitions and therapeutic approaches to metacognition, numerous tools have been developed to assess metacognitive processes in psychosis, each covering different areas (Popolo et al. 2015). In a previously published letter to the editor, we highlighted both the clinical and research needs in this field, stressing the growing number of available instruments and lack of consensus regarding which tools are most appropriate for assessing specific metacognitive domains (Díaz‐Cutraro et al. 2021).

These instruments include tools and interviews that explicitly refer to metacognitive components or processes as well as those that evaluate reflective functions without explicitly mentioning metacognition (Beck et al. 2004; Gutiérrez‐Zotes et al. 2012). Another concern is the growing proliferation of instruments: Alongside tools targeting different metacognitive subdomains, there are now neuropsychological assessments with metacognitive implications as well as clinical and cognitive insight measures. Despite this expansion, there is still no shared framework for determining which tools best assess metacognition or how existing instruments relate to one another. This lack of clarity complicates both the clinical application and the comparison of research findings across studies.

### 1.3 Aims

In light of the aforementioned lack of consensus, the present study aimed to organize the available metacognitive domains and corresponding assessment tools, thereby laying the groundwork for future agreement on a core set of measures.

As a first step toward this goal, we conducted a systematic review of the instruments used to assess metacognition in psychosis. Based on the identified tools, we propose a framework for organizing metacognitive assessments into distinct domains and suggest which instruments appear most suitable for evaluating each of these domains.

## 2 Methods

We followed the guidelines of the Preferred Reporting Items for Systematic Reviews and Meta‐Analyses PRISMA (Page et al. 2021) and registered the study in PROSPERO's International Prospective Register of Systematic Reviews (CRD42020198821) (Diaz‐Cutraro et al. 2024).

### 2.1 Eligibility Criteria

#### 2.1.1 Inclusion Criteria

We included studies that met the following criteria:

1. Use a tool, measure or interview that aims to assess metacognition in the following ways: (a) studies that directly mention that they measure metacognition or (b) studies that measure supra‐ordinary thought processes, emotions and behaviours, which involve processes of reflection on them; or (c) constructs agreed upon by the authors of the manuscript with greater expertise in the definition of metacognition (GD, PL, SM and SO): cognitive insight, self‐regulatory executive function, suppressed thoughts, Meta‐thinking, Reflective Function, Knowledge of mental states, Awareness, Self‐reflectivity/ness and Introspective accuracy.
2. The population assessed in item 1 was diagnosed as suffering from the spectrum of schizophrenia‐related disorders (American Psychiatric Association (APA) 2013) (if the studies compared different samples, they were included when the sample contained 80% or more participants with diagnoses in the spectrum).
3. The paper describes psychometric properties and technical specificities of the measure.
4. Articles are written in English, Spanish, German or Italian.

#### 2.1.2 Specificities of the Searches

In both screening by title and abstract, as well as by full text, we excluded systematic reviews and articles that cited a tool for assessing metacognition but did not meet the requirements established in point 3. In both cases, we tracked the first version of the tool through bibliographic citations and search engines, such as Google Scholar and PubMed, until we found it.

#### 2.1.3 Final Exclusion of Terms

We initially decided to include two search terms that were later excluded because they did not meet the criteria explained in the previous paragraph: mentalizing and emotional intelligence. After an exhaustive review of the literature, both constructs revealed processes close to what has been agreed upon as Social Cognition in several rigorous studies (A. E. Pinkham et al. 2016, 2018), which indicates processing at the object level and not at the meta level. This means that, in contrast to metacognition, both mentalizing and emotional intelligence comprise processes in themselves and are not a reflection on them.

### 2.2 Information Sources

We performed a literature search using the following databases (first search and updated search dates are specified in brackets): PubMed (22/09/2020 and 24/01/2022), APA‐PsychInfo (05/10/2020 and 24/01/2022), Web of Science Core Collection (5/10/2020 and 24/01/2022), Scopus (22/09/2020 and 24/01/2022) and Cibersam‐Banco de Instrumentos (22/09/2020 and 24/01/2022). We searched Google Scholar and PubMed for references using the snowball effect and those arising from articles, as explained in Section 2.1.2. The appendix shows the details of the search criteria for (Appendix A).

### 2.3 Search Strategy

We developed a structured search strategy using key terms, such as cognitive insight, self‐regulatory executive function, thought suppression, metacognition, reflective function, mental state awareness, self‐reflectiveness and introspective accuracy. Additionally, we included terms related to the psychometric properties of tools, such as measurement, inventory, assessment and diagnosis, focusing on schizophrenia, brief psychotic disorder and at‐risk mental states, among others. To maintain the search within the scope of the study, we applied filters to exclude theses, letters to editors and systematic literature reviews. This approach helped minimize irrelevant results and ensured the inclusion of tools that had undergone peer review, either directly or indirectly. Filters were applied to humans, languages (English, German, Italian, and Spanish) and demographic categories such as adolescents (13–18 years), young adults (19–24 years), adults (19–44 years) and middle‐aged to older adults (45+ years) (see Appendix A).

### 2.4 Selection and Data Collection Process

Two independent reviewers screened titles, abstracts and full texts (first search L.D‐C. and M.F‐Q., update L.D‐C. and R.M.; for details, see Appendix https://1drv.ms/w/s!AgePF4zvq9YAgZlejds9GWY0lLWEow?e=oVlom6). In case of doubt, we have included a third reviewer (H.G‐M. or S.O.). The article selection process, including the elimination of duplicate studies, was performed using Rayyan software (Ouzzani et al. 2016).

L.D‐C. oversaw tracking of the first versions of the searched tools. L.D‐C. and M.V‐R. checked that all the tools included were either the first versions of each scale or the first time the adapted and/or translated version was exposed. If these conditions were not met, the first version was traced until it was found in search engines such as Google Scholar, grey literature and doctoral theses.

Based on the final studies included, we synthesized assessment instruments according to the possible processes/subdomains of metacognition explored, where S.O. was consulted on doubts about the scales included.

Once the search results were obtained, we created a preliminary index to categorize the tools based on the outcomes they measured and the specific domains or processes of metacognition they assessed. This included tools that explicitly measured metacognition as well as those that did not label it as such but met the inclusion criteria of this review. Each tool was associated with its corresponding outcome and domain, as defined either by the scale's original authors or by assigning a label closely aligned with the metacognitive process being measured. The index was then reviewed by expert authors, who either confirmed or modified the categorizations as deemed appropriate.

### 2.5 Effect Measures

To meet the two proposed objectives, we organized the results according to the metacognitive domain and outcome assessed by each tool. This allowed us to identify the tools that best aligned with each metacognitive domain, facilitating a clearer understanding of which instruments are the strongest candidates for future research in this area.

### 2.6 Synthesis Methods

In accordance with the clinical and research needs outlined in our previously published letter to the editor (Díaz‐Cutraro et al. 2021), we chose the following items to describe the results: domain, outcome, scale authors or first‐time implementers in psychosis, year of publication, subpopulation, instrument, variant/adaptation (e.g., Spanish adaptation), whether it is an experimental task, clinician‐reported, patient‐reported, number of items and time required (as far as available).

## 3 Results

### 3.1 Study Selection

#### 3.1.1 Flow Chart

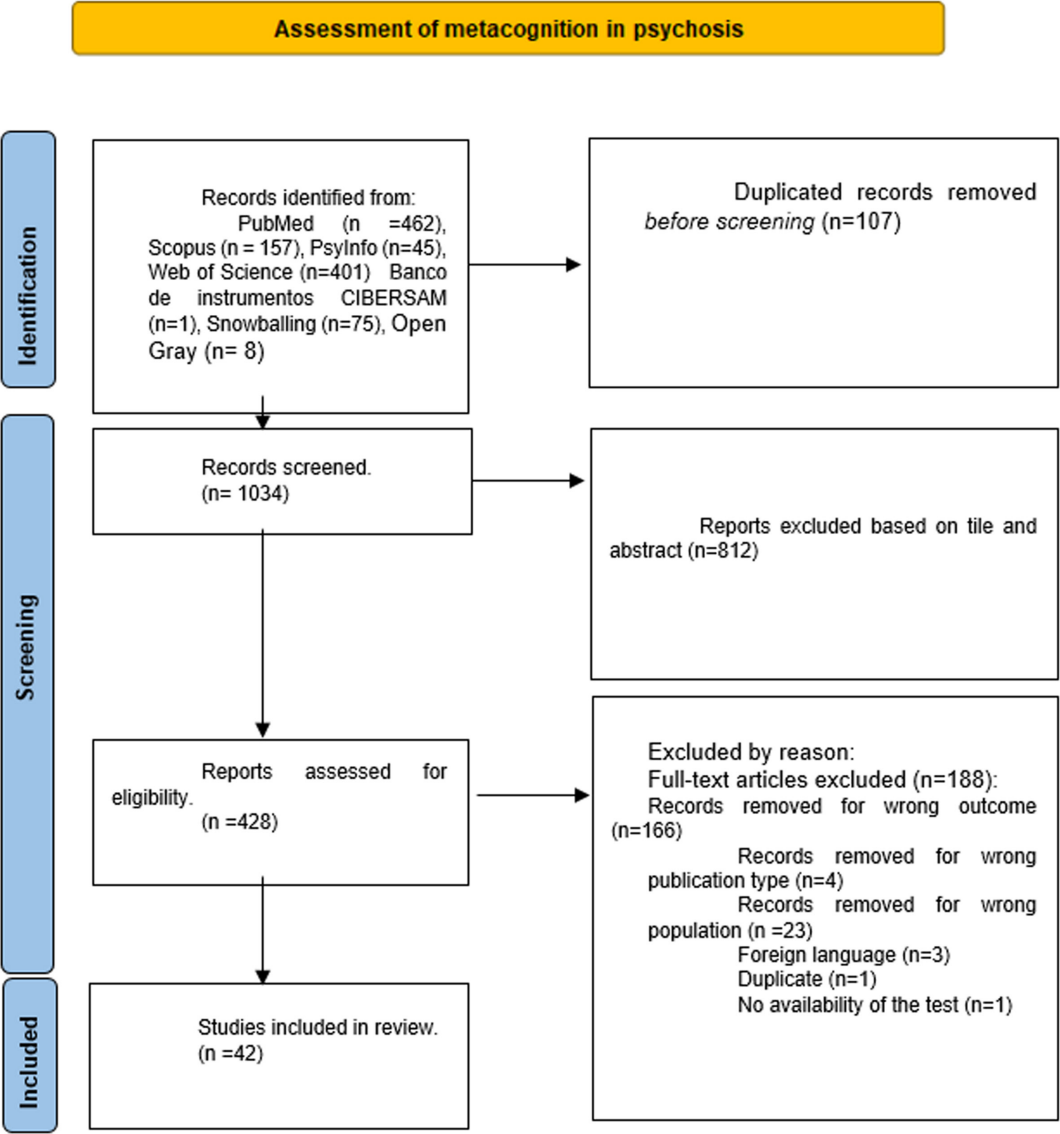

### 3.2 Synthesis of Results

#### 3.2.1 Description of the Measures

We identified 28 questionnaires; Table 1 summarizes the main characteristics of these instruments, including author or implementer, year of first implementation in psychosis, type of instrument (classified according to the Clinical Outcome Assessment framework) (ECNP COA Group Guide 2023), type of measure and number of items.

**TABLE 1:** Description of the measures.

Outcome measured	Author of the assessment tool or implementor for first time in psychosis, version (e.g., Spanish adaptation, first time used in psychosis, etc.)	Instrument name and acronyms	Description	Type of task	Number of items (if applicable)	Time requested
Self‐monitoring for actions	First‐time used in psychosis (Gawęda et al. 2018)	Action memory task (over‐confidence in errors)	Participants received either verbal or nonverbal instructions for actions. Tasks framed in green required physical actions, while those in red were to be imagined. Participants were later asked to recall the actions and judge whether they had been performed or imagined. They also indicated the type of presentation (verbal or nonverbal), rated their confidence and assessed the nature of the action (performed or imagined) with confidence ratings.	PerfO	N/A	N/D
Cognitive insight	French adaptation (Favrod et al. 2008), Taiwanese validation (Kao and Liu 2010), Spanish adaptation (Gutiérrez‐Zotes et al. 2012), Portuguese adaptation (Pinho et al. 2021). All in people with schizophrenia	Beck Cognitive Insight Scale (BCIS)	An instrument that assesses cognitive insight (the ability to attribute meaning to symptoms and awareness of one's own clinical state) and is composed of two subscales, Self‐reflectivity (the ability to introspect and examine one's own thoughts, feelings, and behaviours) and Self‐certainty (the degree of confidence that an individual has about their own beliefs, attitudes or judgements). Psychometric analyses indicate good reliability and validity, with stable factor structures and significant correlations with other measures of insight and psychopathology.	PRO	30 (15‐item version in Tamil)	~5–10 min
Metacognitive beliefs/clinical insight	Original version (Morrison et al. 2005), short‐form (Gumley et al. 2010), short form (Morrison et al. 2011). Non‐clinical population (Morrison et al. 2005; Gumley et al. 2010), schizophrenia (Morrison et al. 2011)	Beliefs about Paranoia Scale (BaPS)	The Beliefs about Paranoia Scale (BaPS) is a self‐report questionnaire designed to measure metacognitive processes related to paranoia by assessing both positive and negative beliefs about it. It consists of a number of attitudes and thoughts that people have expressed about paranoia based on clinical knowledge of patients experiencing persecutory delusions.	PRO	31 (original version), 18 (short form)	~5 min
Unhelpful metacognitive coping strategies	First‐time used in psychosis (Sellers, Wells, and Morrison 2018)	Cognitive Attentional Syndrome Questionnaire (CAS‐1)	The Cognitive‐Attentional Syndrome Questionnaire (CAS‐1) is a short self‐descriptive measure developed to provide information regarding the severity of cognitive‐attentional syndrome (a cluster of cognitive and attentional processes characterized by heightened self‐focused attention, rumination, and worry), a key construct in metacognitive therapy.	PRO	16	5 (ND in the article)
Self‐monitoring	Original version in schizophrenia (Koren et al. 2006), original version in adolescents at ultra‐high risk of psychosis (Scheyer et al. 2014)	Computerized Metacognition Version of the Wisconsin Cars Sorting Test (WCST)	The WCST is a neuropsychological performance‐based task used to assess executive functions, such as cognitive flexibility and abstract reasoning. It has been adapted into computerized versions, including verbal variants, to evaluate metacognitive control and error awareness. It has shown discriminant validity and sensitivity to cognitive change across populations including schizophrenia, aphasia, mild cognitive impairment and substance use disorders.	PerfO	64	N/D
Emotion regulation awareness (suppression and reappraisal)	Possible first‐time use in psychosis–English version (Kimhy et al. 2020), Chinese version (Lam et al. 2022)	Emotion Regulation Questionnaire (ERQ)	The ERQ is a brief self‐report measure assessing the habitual use of two emotion regulation strategies: cognitive reappraisal (modifying thoughts to alter emotional impact) and expressive suppression (inhibiting emotional expressions).	PRO	10	N/D
Metaperception of personality	First version of this combination of assessment tasks (A. E. Pinkham et al. 2019). People with schizophrenia and schizoaffective disorder	Empirical approach to measure tracking accuracy and directional bias (videotaped ‘get to know you’ conversation) + Mini‐IPIP	A multimodal approach combining a video‐recorded social interaction task with the Mini‐IPIP personality inventory to assess metaperception. It evaluates the accuracy and directional bias of individuals' perception of how others see them, by comparing their judgements with observers' ratings.	PerfO, ClinRO, PRO	20 (Mini‐IPIP)	Video: 5 min; Mini‐IPIP: N/D
Metacognitive capacity	First time in psychosis (Lysaker et al. 2016)	Escala de Evaluación de la Metacognición (EEM‐A)	The Metacognition Assessment Scale–Abbreviated version (MAS‐A) evaluates an individual's capacity to think about their own and others' mental states. It has demonstrated good interrater reliability and convergent validity with other metacognitive measures.	ClinRO	N/D	30–60 min
Awareness of reasoning biases	Original version in psychosis (Hardy et al. 2020)	Fast and Slow Thinking (FaST) questionnaire	The FaST is a brief self‐report questionnaire measuring fast and slow thinking patterns associated with paranoid ideation. It has been validated in both clinical and non‐clinical samples.	PRO	12	N/D
Self‐monitoring	First use in general population with psychotic‐like experiences (Reeder et al. 2010)	General Questions Task–Modified Version (GQT)	A modified version of the General Questions Task (GQT) assessing cognitive confidence. Participants answer general knowledge questions and rate their confidence in each response. This allows for the evaluation of confidence accuracy and the exploration of metacognitive processes involved in decision‐making and memory.	PerfO	39	N/D
Emotion regulation awareness	First time in psychosis–schizotypy (Li et al. 2019), German version (Maaßen et al. 2021)	Levels of Emotional Awareness Scale (LEAS)	The LEAS is a performance‐based measure that assesses the ability to identify, interpret and verbalize one's own and others' emotional responses. It has demonstrated excellent internal consistency and high correlation with hand scoring across different languages. LEAS scores are associated with emotion regulation, social functioning, theory of mind and other relevant clinical parameters. It has been used in clinical care and research, including ecological momentary assessment adaptations.	PerfO	20	N/D
Metacognitive abilities	First time used in psychosis (Lysaker et al. 2005), Spanish version in schizophrenia (Lysaker et al. 2016), German version in schizophrenia spectrum disorders (Bröcker et al. 2017)	Metacognition Assessment Scale (MAS‐A)	The MAS‐A is a clinician‐rated instrument designed to assess metacognitive abilities in people with psychosis. It evaluates the capacity to form integrated representations of oneself and others, to use this information to respond to psychosocial challenges and to reflect on one's own mental states. The scale has demonstrated good internal consistency, interrater reliability and construct validity. It has been validated in several languages and clinical contexts.	ClinRO	34	30–60 min
Awareness of cognitive biases	First time used in psychosis (Erawati et al. 2014)	Metacognitive Ability Questionnaire (MAQ)	The MAQ is a self‐report instrument developed to assess metacognitive abilities in individuals with schizophrenia, particularly in relation to their awareness and regulation of cognitive biases. It aims to support clinical insight and reduce misinterpretations by enhancing reflection on thought processes.	PRO	29	N/D
Metacognitive strategies	First time used in psychotic‐like experiences (Reeder et al. 2010), first time in psychosis (Wright et al. 2018)	Metacognitive Assessment Inventory (MAI)	The MAI is a self‐report measure that assesses metacognitive knowledge (awareness of one's own cognitive processes) and metacognitive regulation (monitoring and control of those processes). It has been applied in various contexts, including in individuals with psychotic‐like experiences, and has shown utility for evaluating strategic metacognitive use.	PRO	52	N/D
Metacognitive strategies	Korean population–revised version of the MCQ‐30 with added anger and anxiety components (Han and Lee 2022). People with schizophrenia	Metacognitive Rating Scale (MCRS)	The MCRS is a self‐report questionnaire composed of five subscales: positive beliefs about worry, negative beliefs about uncontrollability and danger of worry, cognitive confidence, need for control and cognitive self‐consciousness. It was developed to assess metacognitive strategies in individuals with schizophrenia and has demonstrated good internal consistency and validity.	PRO	30	N/D
Metacognitive sensitivity and efficiency	First time used in psychosis (Wright et al. 2018)	Metacognitive sensitivity (meta‐d′) and metacognitive efficiency (meta‐d′/d)	Performance‐based indices used to evaluate metacognitive efficiency—the capacity to distinguish between correct and incorrect perceptual decisions relative to actual task performance. Meta‐d′ reflects metacognitive sensitivity, while meta‐d′/d′ offers a normalized efficiency index, widely adopted as a robust measure of perceptual metacognition.	PerfO	N/A	N/D
Mindful attention	First time used in psychosis (FEP) (González‐Blanch et al. 2022)	Mindful Attention Awareness Scale (MAAS)	The MAAS is a self‐report questionnaire that evaluates dispositional mindfulness—specifically, the capacity to attend to and be aware of experiences occurring in the present moment. It has been widely used across diverse populations and settings and has demonstrated robust psychometric properties, including good validity and reliability.	PRO	15	N/D
Metacognitive performance (sensitivity, relative sensitivity, response efficiency)	First time used in psychosis (Muthesius et al. 2021)	Modified version of a Social Perception Task	This performance‐based task evaluates perceptual metacognition in social contexts. Participants judge whether two moving dots are chasing each other and then rate their confidence. The task manipulates perceptual ambiguity using cross‐correlation levels across four trial types. Confidence judgements are used to calculate sensitivity and response efficiency.	PerfO	40 trials (10 per condition)	~4.3 s per trial + confidence rating (self‐paced)
Interoceptive awareness	First time used in the general population with psychotic‐like experiences (Barbato et al. 2021)	Multidimensional Assessment of Interoceptive Awareness‐2 (MAIA‐2)	The MAIA‐2 is a self‐report questionnaire assessing interoceptive awareness across eight subscales: Noticing, Not‐Distracting, Not‐Worrying, Attention Regulation, Emotional Awareness, Self‐Regulation, Body Listening and Trusting. Responses are rated on a 6‐point Likert scale. It has demonstrated strong internal consistency and reliability in both clinical and non‐clinical samples.	PRO	37	N/D
Metacognitive confidence	First time used in psychosis (Szczepanowski et al. 2020)	Post‐Decision Wagering (PDW) task	The PDW task is a performance‐based measure of metacognitive confidence in memory. After making a decision, participants wager imaginary money to indicate how confident they are in the correctness of their response. Higher wagers reflect higher confidence. The task is designed to quantify confidence calibration.	PerfO	N/D	N/D
Metacognitive knowledge and experience	First time used in psychosis (Wright et al. 2018)	Prospective and Retrospective Metacognitive Knowledge	Participants estimate their performance in a cognitive task both before (prospective) and after (retrospective) completing it. These ratings provide insight into metacognitive monitoring accuracy and calibration.	PerfO	N/A	N/D
Emotion regulation strategy (rumination)	First time used in psychosis–Chinese version (Lam et al. 2022)	Short Ruminative Response Scale (SRRS)	The SRRS is a brief self‐report instrument that assesses the frequency of ruminative responses, specifically how often individuals repetitively think about their mood and problems. It is commonly used to measure maladaptive emotion regulation strategies.	PRO	10	N/D
Mindful attention	First time used in psychosis (Chadwick et al. 2008)	Southampton Mindfulness Questionnaire (SMQ)	The SMQ is a self‐report scale designed to assess mindful awareness of distressing thoughts, images and perceptions. Initially conceptualized with four facets, it was later validated as a unidimensional measure. The SMQ has demonstrated strong psychometric properties and has been validated in various populations and languages.	PRO	16	N/D
Meta‐memory evaluation strategy/meta‐memory ability quantification	First time used in psychosis (Zheng et al. 2022	Temporal Order Judgement (TOJ)	A performance‐based task where participants observe dot movements and judge whether one dot is chasing the other, followed by confidence ratings. The task is repeated across three sessions (initial, 2‐h and 24‐h delay) to assess temporal dynamics in metacognitive monitoring.	PerfO	12 stimuli per session	~4.4 min (session 1) + RT; sessions 2 and 3 repeat task without video
Metacognitive strategies	First time used in psychotic‐like experiences (Reeder et al. 2010); first time used in At‐Risk Mental State (Bright et al. 2018)	Metacognitions Questionnaire‐30 (MCQ‐30)	The MCQ‐30 is a self‐report instrument assessing maladaptive metacognitive beliefs central to the metacognitive model of psychopathology. It includes 30 items grouped into five subscales: positive beliefs about worry, negative beliefs about uncontrollability and danger of worry, cognitive confidence, need to control thoughts and cognitive self‐consciousness. It has demonstrated good reliability and validity across different populations, including individuals at risk for psychosis.	PRO	30	N/D
Suppression	First time used in psychosis (FEP), Romanian adaptation (Popa et al. 2021)	White Bear Suppression Inventory (WBSI)	The WBSI is a self‐report questionnaire used to assess the frequency and effectiveness of thought suppression, particularly in response to intrusive or distressing thoughts. It has been applied in various psychological conditions, including psychosis.	PRO	15	N/D
Metacognitive strategies	First time used in schizophrenia (Brunet‐Gouet et al. 2020)	Versailles Metacognitive Strategies Evaluation Questionnaire (V‐MSEQ)	The V‐MSEQ is a self‐report tool that evaluates the use and perceived effectiveness of metacognitive and help‐seeking strategies in individuals with mental disorders. It aims to better understand how individuals approach decision‐making and problem‐solving.	PRO	25	N/D
Adequacy between visual discrimination performance and confidence	First time used in psychosis (Faivre et al. 2021)	Visual Discrimination Task and Confidence Rating	This performance‐based task assesses the ability to discriminate between visual patterns and rate confidence in one's decisions. It evaluates how well individuals with psychosis can judge the accuracy of their perceptual performance.	PerfO	10 blocks of 30 trials	~1 h

*Note:* COA type is categorized according to the *Clinical Outcome Assessment* (COA) framework: PRO (Patient‐Reported Outcome), ClinRO (Clinician‐Reported Outcome), PerfO (Performance Outcome) and ObsRO (Observer‐Reported Outcome). N/A indicates ‘Not applicable’ (e.g., the measure does not consist of a fixed number of items). N/D stands for ‘Not described’ in the original publication.

#### 3.2.2 Overview of Instruments Versions

Table 2 presents the 42 identified versions of the metacognitive instruments, including their authors, year of implementation, version and type of measurement. The Beck Cognitive Insight Scale (BCIS) is among the most widely adapted instruments, with validated versions in Spanish, French, Portuguese and Taiwanese and translations into regional languages such as Kannada and Tamil in India (Beck et al. 2004; Gutiérrez‐Zotes et al. 2012). The Beliefs about Paranoia Scale (BaPS) has also been applied to multiple populations, including non‐clinical groups and individuals with schizophrenia, using both the original and shortened versions (Morrison et al. 2005).

**TABLE 2:** Overview of the 42 versions of instruments used to assess metacognition in psychosis, including authorship, year of implementation, version and COA classification.

Author(s)	Year	Instrument (acronym)	Version/adaptation	COA type
Gawęda et al.	2018	Action Memory Task (Over‐confidence in errors)	First time use in psychosis	PerfO
Gutiérrez‐Zotes et al.	2012	Beck Cognitive Insight Scale (BCIS)	Spanish adaptation and validation	PRO
Favrod et al.	2008	Beck Cognitive Insight Scale (BCIS)	French adaptation and validation	PRO
Pinho et al.	2021	Beck Cognitive Insight Scale (BCIS)	Portuguese adaptation and validation	PRO
Kao and Liu	2010	Beck Cognitive Insight Scale (BCIS)	Taiwanese adaptation and validation	PRO
Jacob et al.	2020	Beck Cognitive Insight Scale (BCIS)	Kannada translation (Indian language)	PRO
Merlin et al.	2012	Beck Cognitive Insight Scale (BCIS)	Tamil adaptation and validation	PRO
Morrison et al.	2005	Beliefs about Paranoia Scale (BaPS)	Original version	PRO
Gumley et al.	2010	Beliefs about Paranoia Scale (BaPS)	Short‐form version	PRO
Morrison et al.	2011	Beliefs about Paranoia Scale (BaPS)	Short‐form version	PRO
Sellers, Wells, Parker, and Morrison	2018	Cognitive Attentional Syndrome Questionnaire (CAS‐1)	First time used in psychosis	PRO
Scheyer et al.	2014	Computerized Metacognition Version of the Wisconsin Card Sorting Test (WCST)	Original version in adolescent population	PerfO
Koren et al.	2006	Computerized Metacognition Version of the Wisconsin Card Sorting Test (WCST)	Original version	PerfO
Kimhy et al.	2020	Emotion Regulation Questionnaire (ERQ)	First use in psychosis	PRO
A. E. Pinkham et al.	2019	Tracking Accuracy & Directional Bias + Mini‐IPIP	First use of this combination of instruments	PerfO + ClinRO + PRO
Lysaker et al.	2016	Escala de Evaluación de la Metacognición (EEM‐A)	First time used in psychosis	ClinRO
Hardy et al.	2020	Fast and Slow Thinking (FaST) questionnaire	First validation in clinical samples	PRO
Reeder et al.	2010	General Questions Task–Modified Version	First time used in general population with psychotic‐like experiences	PerfO
Maaßen et al.	2021	Levels of Emotional Awareness Scale (LEAS)	German version	PerfO
Li et al.	2019	Levels of Emotional Awareness Scale (LEAS)	First time uses in psychosis	PerfO
Lysaker et al.	2005	Metacognition Assessment Scale (MAS‐A)	First time used in psychosis	ClinRO
Bröcker et al.	2017	Metacognition Assessment Scale–Abbreviated (MAS‐A)	German adaptation and validation	ClinRO
Lysaker et al.	2016	Metacognition Assessment Scale–Abbreviated (MAS‐A)	Spanish version	ClinRO
Erawati et al.	2014	Metacognitive Ability Questionnaire (MAQ)	Indonesian adaptation and validation in schizophrenia	PRO
Wright et al.	2020	Metacognitive Assessment Interview (MAI)	First time used of the interview in psychosis	ClinRO
Reeder et al.	2010	Metacognitive Awareness Inventory (MAI)	First time used in general population with psychotic experiences	PRO
Han and Lee	2022	Metacognitive Rating Scale (MCRS)	Korean version with anger and anxiety concepts	PRO
Wright et al.	2019	Metacognitive Sensitivity (meta‐d′) and Efficiency (meta‐d′/d)	First time used in psychosis	PerfO
González‐Blanch et al.	2022	Mindful Attention Awareness Scale (MAAS)	First time used in psychosis	PRO
Muthesius et al.	2021	Modified version of a Social Perception Task	First time used of experimental task in psychosis	PerfO
Barbato et al.	2021	Multidimensional Assessment of Interoceptive Awareness‐2 (MAIA‐2)	First time used in general population with psychotic experiences	PRO
Szczepanowski et al.	2020	Post‐Decision Wagering (PDW) task	First time used in psychosis	PerfO
Lam et al.	2022	Emotion Regulation Questionnaire (ERQ)	Chinese version	PRO
Wright et al.	2019	Prospective and Retrospective Metacognitive Knowledge	First time used in psychosis	PerfO
Lam et al.	2015	Short Ruminative Response Scale (SRRS)	Chinese version and first time used in psychosis	PRO
Chadwick et al.	2008	Southampton Mindfulness Questionnaire (SMQ)	First time used in psychosis	PRO
Zheng et al.	2022	Temporal Order Judgement (TOJ)	First time used of experimental task in psychosis	PerfO
Reeder et al.	2010	Metacognitions Questionnaire‐30 (MCQ‐30)	First time used in general population with psychotic experiences	PRO
Bright et al.	2018	Metacognitions Questionnaire‐30 (MCQ‐30)	First time used in ARMS for psychosis	PRO
Popa et al.	2021	White Bear Suppression Inventory (WBSI)	Romanian version and first time used in psychosis	PRO
Brunet‐Gouet et al.	2020	Versailles Metacognitive Strategies Evaluation Questionnaire (V‐MSEQ)	First time used in schizophrenia	PRO
Faivre et al.	2021	Visual Discrimination Task and Confidence Rating	First time used of experimental task in psychosis	PerfO

*Note:* Instruments are classified according to the Clinical Outcome Assessment (COA) framework: PRO = Patient‐Reported Outcome; ClinRO = Clinician‐Reported Outcome; PerfO = Performance Outcome; ObsRO = Observer‐Reported Outcome. This classification reflects the source of information used to derive the score of each instrument. It helps differentiate self‐assessments from externally observed or performance‐based tools. For instruments combining multiple perspectives (e.g., PRO + PerfO), all applicable COA types are listed.

Other instruments, such as the Metacognition Assessment Scale (MAS‐A) and Metacognitive Assessment Inventory (MAI), have been administered in diverse clinical contexts and provide a structured assessment of metacognitive abilities in psychosis. Some tools were used for the first time in specific populations, such as the General Questions Task–Modified Version in individuals with psychotic‐like experiences (Reeder et al. 2010) and the Mindful Attention Awareness Scale (MAAS) in individuals experiencing a first episode of psychosis (González‐Blanch et al. 2022).

Performance‐based instruments were also identified, including the Computerized Metacognition Version of the Wisconsin Card Sorting Test (WCST) (Koren et al. 2006) for adolescents at ultra‐high risk for psychosis and an Empirical Approach to Measure Tracking Accuracy and Directional Bias in social interactions (A. E. Pinkham et al. 2019). Measures such as the Levels of Emotional Awareness Scale (LEAS) (Lane and Smith 2021) and Southampton Mindfulness Questionnaire (SMQ) (Chadwick et al. 2008) have been validated across languages and cultural contexts.

The broader picture reveals a substantial diversity in tools assessing cognition and metacognition in psychosis. These tools vary not only in their theoretical foundations and targeted constructs but also in their populations of use, psychometric scope and formats, ranging from patient‐reported (PRO) and clinician‐reported (ClinRO) instruments to performance‐based (PerfO) tasks (ECNP COA Group Guide 2023). This heterogeneity underscores the need for a structured classification to clarify what each tool measures.

In the next section, we propose an organization of these instruments by metacognitive domain, with the goal of enhancing conceptual clarity and supporting the selection of the most appropriate tools in both clinical and research settings.

#### 3.2.3 Proposed Metacognition Index

In response to the lack of consensus regarding which specific metacognitive constructs are targeted by each available tool, we propose an organizational framework that groups the instruments into four overarching domains: Metacognitive Awareness, Metacognitive Capacity, Neurometacognition and Social Metacognition (Table 3).

**TABLE 3:** Metacognition index.

Domains of metacognition	Reflection process	Outcome measured	Instrument
Metacognitive awareness	Reflection about thinking styles	Thinking processes	Fast and Slow Thinking Questionnaire (FaST) (Hardy et al.)
	Reflection about reflexive processes	Unhelpful metacognitive coping strategies	Cognitive Attentional Syndrome Questionnaire (CAS‐1) (Sellers et al.)
	Reflection about type of thought content	Cognitive insight	Beck Cognitive Insight Scale (BCIS) (Favrod et al.), Metacognitive Ability Questionnaire (MAQ) (Erawati), Metacognitive Rating Scale (MCRS) (Han and Lee), Beliefs about Paranoia Scale (BaPS) (Morrison et al.)
	Reflection about connection with the present	Mindful attention	Mindful Attention Awareness Scale (MAAS) (González‐Blanch et al.), Southampton Mindfulness Questionnaire (SMQ) (Chadwick et al.)
	Reflection about metacognitive beliefs/beliefs about thinking	Metacognitive strategies	The Metacognitions Questionnaire‐30 (MCQ‐30) (Bright et al.)
	Reflection about metacognitive strategies	Metacognitive strategies	Versailles Metacognitive Strategies Evaluation Questionnaire (V‐MSEQ) (Brunet‐Gouet et al.)
	Reflection about decisions made	Metacognitive knowledge and experience	Prospective and Retrospective Metacognitive Knowledge (Wright et al.)
Metacognitive capacity	Reflection on the self	Metacognitive capacity	Escala de Evaluación de la Metacognición (EEM‐A) (Lysaker et al.)
	Metacognitive skills	Metacognitive abilities	Metacognition Assessment Scale (MAS‐A) (Lysaker et al.)
Neurometacognition	Reflection about performance	Over‐confidence in errors	Action memory task (Gawęda et al.)
		Self‐monitoring	Computerized Metacognition Version of the Wisconsin Card Sorting Test (WCST) (Koren et al.), General Questions Task–Modified Version (Reeder et al.)
		Meta‐memory evaluation strategy/meta‐memory ability quantification	Temporal Order Judgement (TOJ) (Zheng et al.)
		Adequacy between visual discrimination performance and confidence	Visual Discrimination Task and report their confidence (Faivre et al.)
		Metacognitive sensitivity and efficiency	Metacognitive Sensitivity (meta‐d′) and Metacognitive Efficiency (meta‐d′/d) (Wright et al.)
Social metacognition	Reflection about emotional regulation processes (suppression and reappraisal, rumination)	Emotion regulation awareness	Emotion Regulation Questionnaire (ERQ) (Kimhy et al.); Short Ruminative Response Scale (SRRS) (Lam et al.), Levels of Emotional Awareness Scale (LEAS) (Li et al.), The White Bear Suppression Inventory (WBSI) (Popa et al.)
	Reflection about social performance (sensitivity, relative sensitivity, response efficiency)	Metacognitive social performance	Modified version of a social perception task (Muthesius et al.)
	Reflection about biases in social interactions in conjunction with personality traits	Metaperception of personality	Empirical Approach to Measure Tracking Accuracy and Directional Bias (Pinkham et al.)
	Reflection about strategies used in learning	Metacognitive strategies	Metacognitive Assessment Inventory (Reeder et al.)

*Note:* Organization of metacognitive assessment tools based on domains, reflective processes, targeted outcomes and corresponding instruments.

This domain‐based classification offers a conceptual foundation for better understanding the heterogeneity of tools currently used in metacognitive research in psychosis. Although not intended as a definitive model, it provides an initial structure to refine the conceptual boundaries of metacognitive assessment and guide the selection of instruments for both clinical and research purposes.

### 3.3 Excluded Measures

Certain instruments were excluded from this review, even though they were closely related to the search terms, because they did not meet the core inclusion criterion of the presence of a reflective process at a sublevel of thinking, acting or feeling (see Section 2.1). Specifically, we excluded measures that assess clinical insight, introspective accuracy bias (Silberstein et al. 2019; Springfield and Pinkham 2020) and social cognition (Silberstein et al. 2019; Springfield and Pinkham 2020), Popolo et al. (2015).

Regarding **clinical insight**, we initially included several instruments that emerged from the database searches to evaluate whether they assessed processes at the object level (e.g., knowledge about illness) or meta‐level (e.g., reflection on one's own experience of illness). Upon closer examination, however, we found that these measures primarily focused on clinical knowledge, such as awareness of the diagnosis, symptoms or the need for treatment, rather than involving a reflective process about the illness experience itself. Consequently, we excluded them because they did not align with the metacognitive criteria established for this review. Examples of such measures include the *Birchwood Insight Scale (BIS)* (Birchwood et al. 1994; Cleary et al. 2014), the *Awareness* and *Clarity* subscales of the *DERS*, the *Levels of Recovery Scale (LORS)* (Corriveau and Sousa 2013), the Scale to Asses Unawareness of Mental Disorder (SUMD) (Ruiz et al. 2008; Tranulis et al. 2008), the Schedule for Assessment of Insight–Expanded Version (SAI‐E) (Soriano‐Barceló et al. 2016), the Subjective Experience of Deficits in Schizophrenia (SEDS) (Liddle and Barnes 1988), VAGUS Clinician‐Reported (VAGUS‐CR) (de León et al. 2018; Gerretsen et al. 2014), the World Health Organization Disability Assessment Schedule (WHODAS II)–Awareness subscale (World Health Organization 2010) and the Insight and Treatment Attitudes Questionnaire (ITAQ) (Mcevoy et al. 1996).

**Introspective accuracy bias**, which refers to the discrepancy between a person's subjective reports of their motives or abilities and objective reality, was initially considered because empirical evidence suggests that people with schizophrenia may show reduced introspective accuracy, often leading to overconfidence in false memories (Silberstein and Harvey 2019). Although introspective accuracy has been conceptually linked to metacognition (Balzan et al. 2016), the instruments used to assess it in the reviewed studies did not fulfil our inclusion criteria. Specifically, we required that tools explicitly assess a **reflective process** wherein individuals evaluate, monitor or reflect on their own mental states, thoughts, emotions or behaviours. Measures of introspective accuracy typically capture a discrepancy between subjective and objective performance, but they do not involve an individual's awareness of that discrepancy or any active reflection on it (Stip et al. 2003). Therefore, despite their theoretical relevance, these measures were excluded from the review.

## 4 Discussion

The first aim of this study was to conduct a systematic review to identify a broad range of tools, interviews and models used to assess metacognition in psychosis, organizing these instruments according to the categories, constructs, functions and metacognitive goals addressed. The second aim was to explore, based on the instruments identified, the types of outcomes measured and the possible domains of metacognition they targeted in order to propose a preliminary index. To guide our efforts, we adopted a global and inclusive definition of metacognition that encompasses several domains and functions related to the reflection of cognition, emotions and behaviours. This perspective considers metacognition an umbrella term that ranges from immediate awareness of one's mental processes to more complex activities that integrate information into self‐representations.

In line with the first objective, we identified 42 versions of the 31 instruments used to assess metacognition in psychosis. The most frequently used measures were the *Beck Cognitive Insight Scale (BCIS)*, *Beliefs about Paranoia Scale (BaPS)* and *Metacognition Assessment Scale–Abbreviated (MAS‐A)*. The tools reviewed were used in both clinical and research contexts and reflected a wide variety of assessment approaches. Although we adopted an inclusive lens, strict criteria were applied when selecting the final set of tools. Specifically, we included only those measures that aligned with our working definition of metacognition, that is, tools that assess reflective processes, meaning the capacity to explicitly monitor, evaluate or regulate one's own thoughts, emotions or behaviours. This led us to exclude tools assessing clinical insight when they focused solely on illness‐related knowledge or awareness at the object level (e.g., knowing one has a diagnosis) without incorporating metacognitive reflection on that knowledge.

Regarding the second aim, our content analysis enabled us to group the instruments into four overarching domains of metacognition, each defined by the type of reflective process assessed. Metacognitive Awareness encompasses reflection on thinking styles, types of cognitive content and beliefs about one's thinking. Metacognitive Capacity refers to the ability to form and use complex integrated representations of oneself and others. Neurometacognition involves the reflection of neurocognitive functioning and performance. Finally, Social Metacognition includes reflections on emotional understanding, social skills, and interpersonal biases. This categorization aligns with previous characterizations (e.g., A. E. Pinkham 2019), which distinguish between discrete metacognitive processes, such as basic monitoring of specific cognitive phenomena, and integrated processes that involve synthesizing diverse mental contents into broader representations of the self and others.

In addition, we identified two complementary frameworks for classifying metacognitive assessments. The first is a process‐based approach that places instruments along a gradient from discrete to integrative mental operations. Some tools focus on specific deficits, such as memory biases or autobiographical reasoning, whereas others assess more complex, higher order reflective capacities, including the ability to construct a coherent self‐narrative. The second is a domain‐based approach that emphasizes the presence or absence of reflective processes within specific domains such as cognition, emotion and social interaction.

Together, these two perspectives offer a more nuanced understanding of how metacognition is assessed in psychosis and provide criteria to guide the selection of appropriate instruments, depending on the specific goals of evaluation. This distinction has practical implications in both the clinical and research contexts. In time‐limited clinical settings, brief and self‐administered tools such as *BCIS* (Beck et al. 2004) or *MCQ‐30* (Wells and Cartwright‐Hatton 2004) may be particularly useful. Conversely, settings that allow for more extensive assessments can benefit from integrative instruments such as the *EEM‐A* in combination with the *MAS‐A*. Experimental paradigms, including confidence‐based tasks, such as the *Post‐Decision Wagering Task* or the metacognitive version of the *Wisconsin Card Sorting Test*, may be more suitable for research environments.

From a therapeutic perspective, this classification also resonates with the principles shared by third‐wave cognitive‐behavioural therapies and process‐based approaches. These models emphasize components such as emotion regulation, mindfulness, acceptance and self‐awareness and view therapeutic change as involving shifts in metacognitive functioning. Current psychotherapeutic approaches for psychosis, including *Metacognitive Therapy* (Morrison et al. 2014), *Metacognitive Training (MCT)* (Moritz and Woodward 2007), *MERIT* (P. H. Lysaker and Klion 2017), and *Metacognitive Interpersonal Therapy (MIT‐P)* (Salvatore et al. 2018)—integrate these principles and stress the importance of tailoring interventions to the individual's specific metacognitive profile. In this regard, our proposed index may help clinicians to identify relevant domains for therapeutic intervention and select appropriate tools to support treatment planning and progress monitoring.

By organizing metacognitive domains in psychosis, we offer a framework that facilitates a better understanding of the diversity of the reflective processes involved, their relevance to clinical symptoms and functioning, and their role as potential therapeutic targets. The results presented here, along with the proposed tables and index, aim to support a more precise and goal‐oriented selection of assessment tools in both clinical and academic settings.

### 4.1 Limitations

This study had several limitations that should be acknowledged. First, we did not include a formal expert panel in the development of the proposed classification or metacognitive index. Although multiple authors on the team brought specialized expertise in psychosis and metacognition, future work would benefit from the use of structured methodologies such as Delphi or RAND panels to strengthen the validity and consensus behind the categorization. Second, although the review process involved independent evaluations of the instruments by several team members at different time points to ensure consistency, we did not conduct a formal thematic or qualitative analysis using dedicated software. Such an approach could have revealed latent patterns or conceptual groupings that were not readily apparent in the narrative synthesis. Third, the instruments identified vary widely in terms of design, population, intended use, and theoretical orientation. This heterogeneity may limit direct comparability across tools, particularly when considering measurement precision or generalizability. However, given that the goal of this work was to provide a first step toward organizing the existing landscape of metacognition assessment in psychosis, not to establish a definitive classification, we believe that the findings offer a valuable foundation for further refinement and validation (Fitch et al. 2001; A. E. Pinkham et al. 2014).

### 4.2 Further Directions

Future studies should build on the findings of this review by conducting systematic evaluations of each instrument's psychometric properties, clinical utility and risk of bias. These evaluations can support the development of a formal recommendation index to guide instrument selection across different clinical and research contexts. Simultaneously, expert consensus methods such as RAND or Delphi panels can be employed to validate and refine the proposed metacognitive domains. Additional quantitative analyses, including factor analysis or network modelling, may help clarify the latent structure of the proposed index and its relationship to adjacent constructs, such as social cognition, emotion regulation or insight. Longitudinal studies could also explore the sensitivity of different instruments to therapeutic changes and functional outcomes, helping determine which domains are most relevant across the course of psychosis. Together, these lines of work could significantly enhance both conceptual clarity and practical application of metacognitive assessment (Arbuzova et al. 2023; Lund et al. 2023).

## 5 Conclusions

This systematic review offers a structured and integrative overview of instruments used to assess metacognition in psychosis. By organizing tools based on their reflective focus and outcome domains and by identifying four overarching categories—Metacognitive Awareness, Metacognitive Capacity, Neurometacognition and Social Metacognition—this study provides a foundation for consensus building and future refinement.

The proposed metacognitive index is intended to support clinicians and researchers in selecting the most appropriate instruments according to their specific goals, available resources and target population. From a translational perspective, this study highlights the potential of metacognition as a clinical target and offers concrete tools to guide assessment, treatment planning and monitoring of change, especially within third‐wave and integrative psychotherapeutic approaches.

Ultimately, this review aims to move the field closer to a shared understanding of how metacognition can be reliably and meaningfully measured during psychosis. Advancing toward greater consensus in terminology, domains and methodology will be essential for improving both research precision and personalization of care for individuals experiencing psychosis.

## Funding

Luciana Díaz‐Cutraro received a Predoctoral Training Grant in Health Research under this project, awarded by the Instituto de Salud Carlos III (Government of Spain) through research grant FI19/00062 (support for the hiring of predoctoral researchers). She also received funding through the Movilidad de personal investigador contratado en el marco de la AES (M‐AES) program, granted by the Instituto de Salud Carlos III (grant MV20/00019, 2021), which allowed her to work with Dr. Steffen Moritz on the theoretical foundations of this project.

## Disclosure

The present project is registered in the PROSPERO International Prospective Registry of Systematic Reviews under the number CRD42020198821.

## Conflicts of Interest

Dr. Steffen Moritz was the developer of Metacognitive Training (MCT) for psychosis, and Dr. Paul Lysaker was the developer of the MERIT approach for psychosis. However, as this systematic review focuses exclusively on synthesizing instruments used to assess metacognition in psychosis and does not evaluate psychotherapeutic models, we declare no conflicts of interest related to the present work. Dr. Giancarlo Dimaggio is one of the developers of the original *Metacognition Assessment Scale (MAS)* and contributed to the development of the *MAS‐A*. He is also the principal developer of Metacognitive Interpersonal Therapy (MIT) and co‐creator of the MOSST intervention. These roles are acknowledged here for transparency; however, they do not influence the methodology or conclusions of the present study.

## Data Availability

The data that support the findings of this study are available from the corresponding author upon reasonable request.

## Appendix A

### Databases and Search Dates

A systematic literature search was conducted in the following databases: PubMed, APA PsycINFO, Web of Science Core Collection, Scopus, and the CIBERSAM Instrument Bank. Searches were performed twice, initially between September and October 2020 and updated in January 2022. Additional references were identified through snowballing techniques using Google Scholar and PubMed.

### Search Strategy

The search strategy was designed to identify instruments assessing metacognitive processes in psychosis, with a specific focus on reflective processes at the meta‐level. Three core components were combined:

1. Metacognitive constructs,
2. Assessment and psychometric properties, and
3. Psychosis‐spectrum populations.

Equivalent strategies adapted to the syntax of each database were applied.

PubMed

(

metacog*[TIAB] OR

metacognition[TIAB] OR

"metacognitive beliefs"[TIAB] OR

"metacognitive knowledge"[TIAB] OR

"metacognitive function*"[TIAB] OR

"cognitive insight"[TIAB] OR

mentalizing[TIAB] OR

"self‐regulatory executive function"[TIAB] OR

"thought suppression"[TIAB] OR

"meta‐thinking"[TIAB] OR

"knowledge of mental states"[TIAB] OR

awareness[TIAB] OR

self‐reflectiv*[TIAB] OR

"introspective accuracy"[TIAB] OR

"emotional intelligence"[TIAB]

)

AND

(

assessment*[TIAB] OR

evaluation*[TIAB] OR

measurement*[TIAB] OR

instrument*[TIAB] OR

inventory[TIAB] OR

scale*[TIAB] OR

questionnaire*[TIAB] OR

interview*[TIAB] OR

psychometric*[TIAB] OR

reliability[TIAB] OR

validity[TIAB]

)

AND

(

psychosis[TIAB] OR

schizophrenia[TIAB] OR

"schizoaffective disorder"[TIAB] OR

"schizophreniform disorder"[TIAB] OR

"brief psychotic disorder"[TIAB] OR

"delusional disorder"[TIAB] OR

"early psychosis"[TIAB] OR

"first episode psychosis"[TIAB] OR

"at‐risk mental state"[TIAB]

)

NOT

(

meta‐analysis[TIAB] OR

review*[TIAB] OR

treatment*[TIAB] OR

clinical trial*[TIAB] OR

letter*[PT]

)

Filters applied: Humans; languages (English, Spanish, German, Italian); age groups (adolescents, young adults, adults, and middle‐aged to older adults).

APA PsycINFO

(

metacognit* OR

"cognitive insight" OR

mentali* OR

reflectiv* OR

awareness OR

introspective* OR

"emotional intelligence"

)

AND

(

psychosis OR

schizop* OR

"first episode psychosis" OR

"at‐risk mental state" OR

ARMS

)

AND

(

assessment* OR

evaluation* OR

measurement* OR

instrument* OR

inventory* OR

scale* OR

questionnaire* OR

interview* OR

psychometric* OR

reliability OR

validity

)

NOT

(

meta‐analysis OR

review* OR

treatment* OR

clinical trial* OR

letter*

)

Web of Science Core Collection

(

"metacognition" OR

"metacognitive beliefs" OR

"metacognitive knowledge" OR

"metacognitive function*" OR

"cognitive insight" OR

mentalizing OR

"self‐regulatory executive function" OR

"thought suppression" OR

"meta‐thinking" OR

"reflective function" OR

"knowledge of mental states" OR

awareness OR

"self‐reflectivity" OR

"self‐reflectiveness" OR

"introspective accuracy" OR

"emotional intelligence"

)

AND

(

assessment OR

evaluation OR

measurement OR

inventory OR

interview OR

scale OR

questionnaire OR

psychometric OR

reliability OR

validity

)

AND

(

psychosis OR

schizophrenia OR

"early psychosis" OR

"first episode psychosis" OR

"at‐risk mental state"

)

NOT

(

meta‐analysis OR

review* OR

treatment OR

clinical trial* OR

letter*

)

Indexes searched: SCI‐EXPANDED, SSCI, A&HCI, CPCI‐S, CPCI‐SSH, BKCI‐S, BKCI‐SSH, ESCI, CCR‐EXPANDED, IC.

Time period: all years.

### Clarification Regarding Excluded Constructs

Although the initial search strategy included broad terms such as mentalizing and emotional intelligence, these constructs were excluded during title/abstract and full‐text screening. This decision was based on their predominant conceptualization as object‐level social cognition processes rather than meta‐level reflective processes, in accordance with the metacognitive framework adopted in the present review.
